# RNA-Seq-Based Transcriptome Analysis of Aflatoxigenic *Aspergillus flavus* in Response to Water Activity

**DOI:** 10.3390/toxins6113187

**Published:** 2014-11-21

**Authors:** Feng Zhang, Zhenni Guo, Hong Zhong, Sen Wang, Weiqiang Yang, Yongfeng Liu, Shihua Wang

**Affiliations:** 1 Key Laboratory of Pathogenic Fungi and Mycotoxins of Fujian Province, Key Laboratory of Biopesticide and Chemical Biology of the Education Ministry, and School of Life Sciences, Fujian Agriculture and Forestry University, Fuzhou 350002, China; E-Mails: fzhang@fafu.edu.cn (F.Z.); joancikwok@hotmail.com (Z.G.); zhonghong_321@yahoo.com (H.Z.); wangsen123jay@yahoo.com (S.W.); ywq20011@outlook.com (W.Y.); 2 Shenzhen Key Laboratory of Bioenergy, BGI-Shenzhen, Shenzhen 518083, China; E-Mail: liuyongfeng@bgitechsolutions.com

**Keywords:** RNA-Seq, transcriptome, *Aspergillus flavus*, water activity, aflatoxin

## Abstract

*Aspergillus flavus* is one of the most important producers of carcinogenic aflatoxins in crops, and the effect of water activity (a_w_) on growth and aflatoxin production of *A. flavus* has been previously studied. Here we found the strains under 0.93 a_w_ exhibited decreased conidiation and aflatoxin biosynthesis compared to that under 0.99 a_w_. When RNA-Seq was used to delineate gene expression profile under different water activities, 23,320 non-redundant unigenes, with an average length of 1297 bp, were yielded. By database comparisons, 19,838 unigenes were matched well (*e*-value < 10^−5^) with known gene sequences, and another 6767 novel unigenes were obtained by comparison to the current genome annotation of *A. flavus*. Based on the RPKM equation, 5362 differentially expressed unigenes (with |log_2_Ratio| ≥ 1) were identified between 0.99 a_w_ and 0.93 a_w_ treatments, including 3156 up-regulated and 2206 down-regulated unigenes, suggesting that *A. flavus* underwent an extensive transcriptome response during water activity variation. Furthermore, we found that the expression of 16 aflatoxin producing-related genes decreased obviously when water activity decreased, and the expression of 11 development-related genes increased after 0.99 a_w_ treatment. Our data corroborate a model where water activity affects aflatoxin biosynthesis through increasing the expression of aflatoxin producing-related genes and regulating development-related genes.

## 1. Introduction

*Aspergillus flavus*, a widely distributed saprophyte, is the second leading cause of aspergillosis infection in humans and is the leading agent of chronic sinonasal infection in immunocompetent patients [1]. *A. flavus*, which is also an important soil fungus producing highly carcinogenic aflatoxins (AFs), causes damage to different seedcrops, such as corn, cotton, peanuts and tree nuts, both before and after harvest*.* [2,3]. Structurally, the aflatoxins are highly substituted coumarins that contain a fused dihydrofurofuran moiety. The major AFs of concern in nature are designated as B_1_, B_2_, G_1_, and G_2_ [4]. However, among them, AFB_1_ is considered the most predominant, most toxic and most potent hepatocarcinogenic natural compound ever characterized [5]. It has been previously reported that AFB_1_ is produced by strains of *A. flavus* isolated from the corneal material of patients, commodities, and soils [6,7,8]. Mapping of overlapping cosmid clones of *A. flavus* genomic DNA established that the genes in the aflatoxin biosynthetic pathway are clustered and consist of 25 genes spanning approximately 70 kb [9]. Among them, Schmidt-Heydt* et al.* [10] clearly reported that the ratio of *aflR vs.* *aflS* affected aflatoxin pathway gene expression.

AF biosynthesis is regulated by many factors, one of which is environmental cues, including temperature, water activity and pH [11,12]. Although fungal growth is influenced by several environmental factors, the major etiological determinants of fungal growth and mycotoxin production are water activity (a_w_) and temperature [13,14]. Although many reports have profiled the transcriptomes of fungi under different temperatures, no report has yet addressed transcriptome analysis for fungi under water stress [15]. The a_w_ is a measure of the amount of freely available water in an environment for microbial growth and is related to pure water, which has an a_w_ of 100 percent relative moisture [16]. In a recent study, the influence of temperature and water activity on aflatoxin gene expression and phenotypic production of *A. flavus* was analyzed, and it could be demonstrated that a_w_ was the leading parameter [17]. More freely available water induced more sporulation and better growth for *A. flavus*, as well as for other *Aspergillus* species, such as *A. niger* [18]. This phenomenon has been reported in the recent increasing number of studies dealing with the effect of water activity on microbial growth [14,17]. However, there are only a few studies reporting on the impact of water activity on *A. flavus* growth and toxin production using a sys tems approach [16].

Very recently, RNA sequencing (RNA-Seq), a high-throughput and high-resolution sequencing technology, has achieved widespread consideration as a revolutionary tool for transcriptomics study [15,19]. In this study, the RNA-Seq approach was adopted to provide a comprehensive view of the *A. flavus* transcriptome as well as specific data regarding differentially expressed genes between 0.93 a_w_ and 0.99 a_w_. This work improves the understanding of the effect of water activity on development and aflatoxin biosynthesis of *A. flavus* at the transcriptome level. These findings are significant for predicting the impact of climate change on aflatoxin production, which might be used to improve food safety and to develop specific approaches to control such carcinogenic natural metabolites in the food chain.

## 2. Materials and Methods

### 2.1. Fungal Strains and Growth Conditions

The *A. flavus* NRRL 3357 was kindly provided by Zhumei He (Sun Yat-sen University, Guangzhou, China). The strains were inoculated in YES medium (20 g yeast extract, 150 g sucrose, 1 g MgSO_4_·7H_2_O, 1 L). Spores from a 7-day-old culture grown at 37 °C were dislodged with a sterile loop and placed into 10 mL of sterile water +0.05% DMSO in a 25 mL universal bottle. The spores were counted, and a 10^6^ spore mL^−1^ concentration was prepared. The agar medium was modified with glycerol to adjust the water availability to 0.93 a_w_ and 0.99 a_w_, and the following amounts were used per liter (108 mL, 0.99; 24.5 mL, 0.93) [17]. The 9 cm Petri plates containing media treatments were all overlaid with sterile 8.5 cm disc cellophanes and then centrally inoculated with a 10-μL-spore suspension. Replicates (five per treatment) were incubated at 28 °C.

### 2.2. Growth Assessment and Aflatoxin Analysis

After incubation at a different a_w_ level, the colony morphology was observed after 5 days. For quantitative comparison of conidia production, conidia were washed off the agar plates using 0.05% DMSO solution and counted on a hemocytometer. Quantitative determination of aflatoxin B_1_ from fungal colonies was performed by TLC analysis. For this purpose, the biomass was removed from the cellophane surface for aflatoxin extraction. Extraction was performed using 40 mL of chloroform (twice with 20 mL each), and then the chloroform phase was filtered through filter paper and concentrated to dryness under 50 °C in an incubator. The residue was redissolved in 20 μL of methanol, and 10 μL of this solution was spotted and developed on a Si250 silica gel plate (Haiyang, Qingdao, China) with a solvent system of chloroform/acetone (90:10, *v*/*v*) [20]. Aflatoxin production was measured in micrograms per gram of culture biomass.

### 2.3. cDNA Preparation and Illumina Sequencing

Five day-old mycelium was removed from the cellophane surface for isolation of RNA, and cDNA was prepared according to a protocol with some modifications [21]. Genomic DNA was digested using DNase (New England Biolabs, Beijing, China), and total RNA was isolated using TRIzol reagent (Invitrogen, Shanghai, China). A Nano Drop 2000 and Agilent 2100 were used to evaluate the quality of RNA. After total RNA extraction and DNase I treatment, magnetic beads with oligo (dT) were used to isolate mRNA. Mixed with the fragmentation buffer, the mRNA was cleaved into short fragments. Then cDNA was synthesized using the mRNA fragments as templates. Short fragments were purified and resolved with elution buffer for end reparation and single nucleotide adenine addition. After that, the short fragments were connected with adapters. The suitable fragments were selected as templates for PCR amplification. During the QC steps, Agilent 2100 Bioanaylzer and ABI StepOnePlus Real-Time PCR System were used in quantification and qualification of the sample library. Lastly, the library was sequenced using an IlluminaHiSeqTM 2000.

### 2.4. Clean Reads and Sequence Assembly

Raw reads were filtered to remove adaptors, reads with more than 5% unknown nucleotides, and other low quality reads. After QC filtering, the following analysis was performed. Transcriptome *de novo* assembly was conducted with the short-reads assembly program Trinity. Trinity, including three independent software modules, Inchworm, Chrysalis, and Butterfly, was applied sequentially to process large volumes of RNA-seq reads. Trinity partitions the sequence data into many individual de Bruijn graphs, which represent the transcriptional complexity at a given gene or locus. Then, each graph was independently processed to extract full-length splicing isoforms and to tease apart transcripts derived from paralogous genes. The result sequences from Trinity are called unigenes.

### 2.5. Annotation and Analysis of Unigenes

BLASTx alignment (*e*-value < 10^−5^) between unigenes and protein databases, including Nr, Swiss-Prot, KEGG, and COG, was performed, and the best alignment results were used to decide sequence direction of unigenes. If results of different databases conflicted with each other, a priority order of Nr, Swiss-Prot, KEGG, and COG was followed. When a unigene happened to be unaligned to none of the above databases, ESTScan (http://estscan.sourceforge.net) [22], a program that can detect coding regions in low-quality sequences, was introduced to decide its sequence direction. To obtain protein functional annotation, unigenes were aligned by BLASTx to protein databases (*e*-value < 10^−5^), and aligned by blastn to nucleotide databases nt (*e*-value < 10^−5^), retrieving proteins with the highest sequence similarity with the given unigenes. With Nr annotation, the Blast2GO program was used to obtain GO annotation of unigenes. After obtaining GO annotation for every unigene, WEGO software was used to perform GO functional classification for all unigenes and to understand the distribution of gene functions [23]. With the help of KEGG database, we could further study the genes’ biological complex behaviors, and using KEGG annotation we could obtain pathway annotation for unigenes.

### 2.6. Identification and Analysis of Differentially Expressed Genes

First, the RPKM method was used to calculate the expressed value of genes (Reads Per kb per Million reads). The RPKM method is able to eliminate the influence of different gene length and sequencing level on the calculation of gene expression. Therefore the calculated gene expression can be directly used for comparing the different expression between samples. Then, the *p* value was applied to determine differentially expressed unigenes. FDR (False Discovery Rate) control is a statistical method used in multiple hypothesis testing to correct for *p*-value. In our analysis, we choose those with FDR ≤ 0.001 and a ratio ≥ 2. Finally, differentially expressed genes (DEGs) were then subjected to GO functional analysis and KEGG pathway analysis.

### 2.7. Availability of Supporting Data

The raw Illumina sequencing dataset of *Aspergillus flavus* was submitted to the NCBI Sequence Read Archive under the accession number of SRP034649.

## 3. Results and Discussion

### 3.1. Effect of Water Activity on Growth and Aflatoxin Production of A. flavus

**Figure 1:**
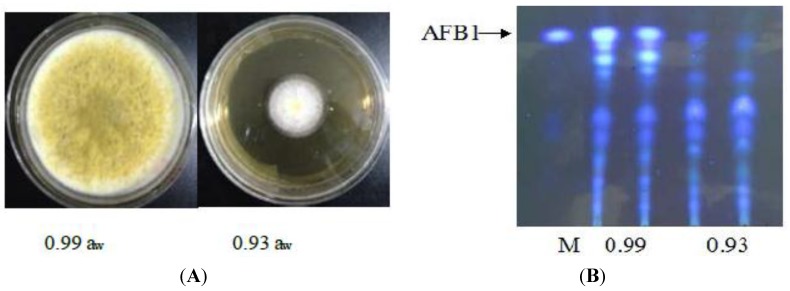
Effect of water activity on *A. flavus* growth and aflatoxin production. (**A**) Representative pictures of a colonial morphology from *A. flavus* at 0.99 a_w_ (left) and at 0.93 a_w_ (right). Strains were incubated at 37 °C for five days; (**B**) Extracts of the *A. flavus* grown for five days on YES medium. Extracts and aflatoxin standards were spotted onto silica gel TLC plates. The plates were visualized under 310-nm UV light.

Growth and aflatoxin production by *A. flavus* at the phenotypic level was monitored in relation to changes in different treatments. As seen in Figure 1A, the colony diameter of strains at 0.93 a_w_ was significantly smaller than that at 0.99 a_w_. When grown on YES plates at 37 °C, the strains under 0.93 a_w_ exhibited decreased conidiation compared to that under 0.99 a_w_, and the strains under 0.93 a_w_ displayed an approximately 16-fold conidia reduction compared with the strains under 0.99 a_w_ (data not shown). As previously described, growth was highly influenced by water activity [24]. To identify the effect of different water activity on aflatoxin production, thin-layer chromatography analysis was performed with the standards of aflatoxin on the silica gel G plates, and the results are shown in Figure 1B. When a_w_ was reduced, there was a sharply decrease in aflatoxin biosynthesis although the culture condition remained at 28 °C. Compared with that at 0.99 a_w_, aflatoxin production was very low, and only other extracted metabolites were observed at 0.93 a_w_. Our findings are consistent with a previous report that more aflatoxin was produced under 0.99 a_w_ than under 0.93 a_w_ [16]. The data indicates that aflatoxin production of *A. flavus* was obviously affected by water activity. This phenomenon may be due to complex regulation of the aflatoxin biosynthesis gene cluster of *A. flavus* in relation to various levels of water activity [17].

### 3.2. Illumina Sequencing and Reads Assembling

**Figure 2:**
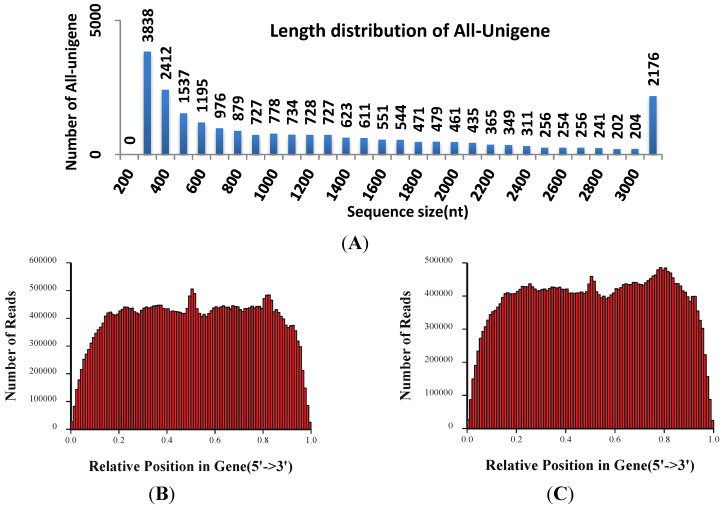
Length distribution and quality-control analysis of RNA-Seq data. (**A**) Length distribution of assembled unigenes; The length of unigenes ranged from 100 bp to over 3000 bp. The total read coverage along the gene body from 5' to 3' end in 0.99 a_w_ (**B**) and 0.93 a_w_ (**C**).

Two cDNA libraries were prepared at the fifth day and sequenced using the Illumina platform. Illumina sequencing generated a total of 41,004,372 reads (0.99 a_w_) and 40,712,492 reads (0.93 a_w_) that were 90 bp in length after stringent data cleaning and quality checks. The mean of Q20 percentage (proportion of nucleotides with quality value larger than 20 in reads), N percentage (proportion of unknown nucleotides in clean reads) and GC percentage are 96.54%, 0.00% and 52.96%, respectively. Trinity was used to assemble clean reads, producing a total of 60,039 contigs with a minor of N50 of 1623 nt (*i.e*., the median length of all unigenes) for *A. flavus*. After further processes of sequence splicing and redundancy removing, a total of 23,320 non-redundant unigenes were identified. Of these, 24,991 and 25,190 unigenes were generated from the 0.99 a_w_ and 0.93 a_w_ treatments, respectively (Table 1). The length distribution in Figure 2A indicated that 47.08% unigenes (total 10,978 unigenes) had a length > 1000 nt (mean 1297 nt).

To evaluate the quality of RNA-Seq data, several quality control analyses were performed. Firstly, the ratio of the gap length of assembled unigenes was assessed, and the results indicate that gap lengths were less than 5% of the total length. In addition, the total coverage of reads from the 5' to the 3' end of genes was examined, and it revealed that both the 0.99 a_w_ and 0.93 a_w_ RNA-Seq reads were evenly distributed with the exception of the very 5' and 3' ends (Figure 2B,C). Therefore, the assembled data are of high quality in current study. The *Aspergillus* genus are widely distributed molds in the environment, many of which are documented to cause human disease [25]. Some of the RNA-Seq data for *Aspergillus* has been published previously [26,27,28], and very recently, Chang* et al.* (2014) compared the different transcriptome profiles of *A. flavus* exposed and not exposed to decanal [29]. To our knowledge, this study was the first report on the complete transcriptome of *Aspergillus* in response to two different water activities using an Illumina paired-end sequencing strategy.

**Table 1:** Summary of RNA-Seq data sets.

Category	Treatments	Total number	Mean length (Nt)	N50
Contigs	0.93 a_w_	29,420	663	1705
	0.99 a_w_	30,619	653	1623
Unigenes	0.93 a_w_	25,190	1004	1740
	0.99 a_w_	24,991	1073	1829

### 3.3. Annotation and Analysis of All-Unigenes

**Figure 3:**
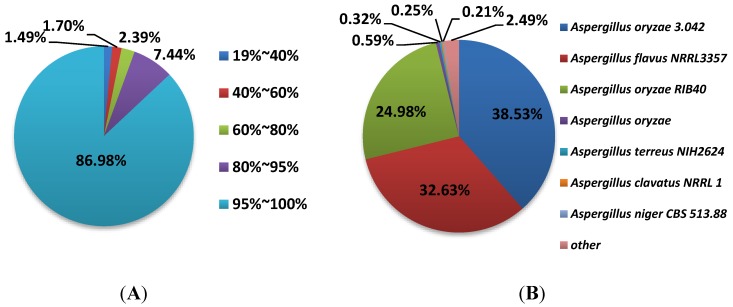
Overview of all-unigene in the *A. flavus* transcriptome. (**A**) the similarity and (**B**) species distribution of all-unigene.

To understand the transcriptome of *A. flavus*, all unigenes were aligned against sequences from the NCBI non-redundant (nr) protein database by using the BLASTx algorithm with an *e*-value threshold of 10^−5^. BLASTx alignment analysis indicated that a total of 19,838 unigenes matched to known proteins in the Nr databases. Thus far, a total of 13,071 genes encoding proteins have already been annotated in the genome of *A. flavus* [30], whereas Lin* et al.* (2013) estimated that *A. flavus* has 14,510 genes by combining NCBI database with their RNA-Seq data [19]. However, the RNA-seq data presented in this work implies that more unigenes have the potential for translation into functional proteins, which serves to enrich the annotation of the *A. flavus* genome. A possible explanation for this phenomenon is posttranscriptional regulation, such as alternative splicing and RNA editing, enlarges their transcripts diversity [21]. Figure 3A,B show the similarity distribution of all unigenes in detail. The results indicate that 96.81% of all unigenes had an identification of more than 60% of the annotated genes. In a comparison with the nr database, we interestingly found that 38.5% of sequences matched to that of *A. oryzae*, but only 32.6% unigenes were well matched to that of *A. flavus.* However, Yu* et al.* (2008) found *A. oryzae* shared over 95% identity to *A. flavus* on the DNA level, and fewer than 300 genes were unique to each species [29].

### 3.4. Functional Analysis and Classification of All-Unigenes

**Figure 4:**
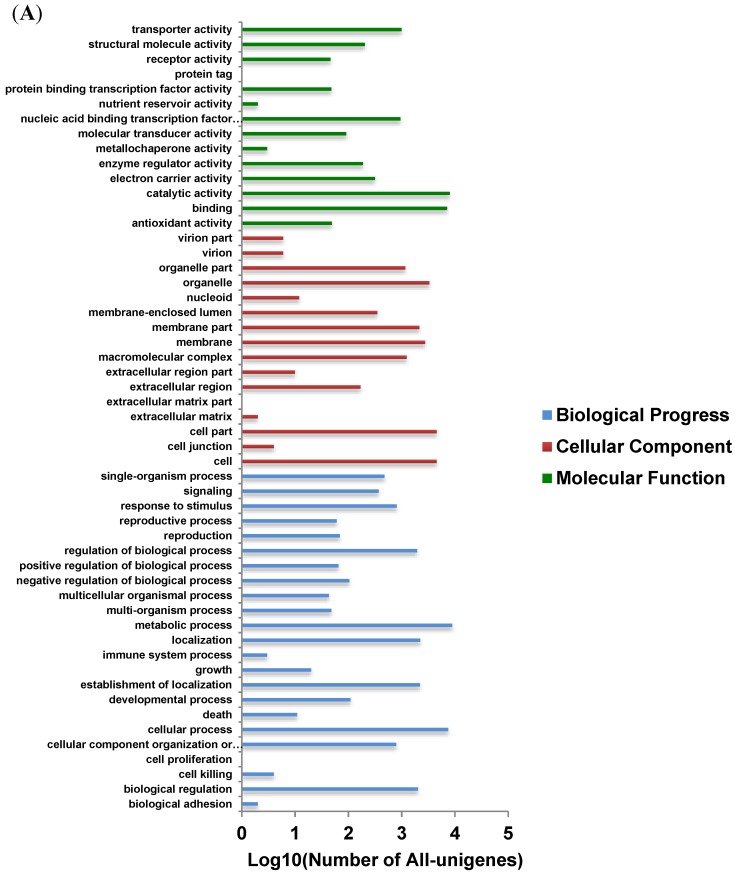

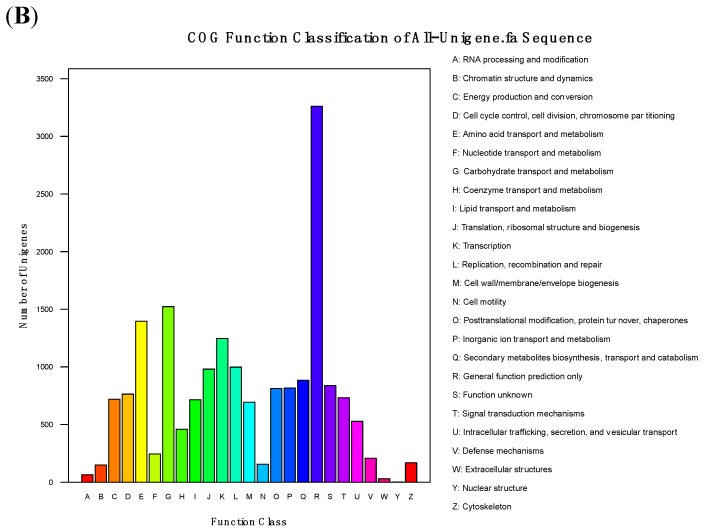
Annotation of all-unigene in the *A. flavus* transcriptome. (**A**) The gene ontology annotation of all-unigene; (**B**) Histogram presentations of clusters of orthologous groups (COG) classification.

To deeply understand the transcriptome of *A. flavus*, GO (Gene Ontology) and COG (Clusters of Orthologous Groups of proteins) were applied to classify functions of the predicted all unigenes. A total of 13,342 unigenes were grouped to at least one GO term, and these unigenes were classified into three functional categories (Figure 4A). Sequences with GO terms corresponding to the “biological process” group were divided into 23 subcategories, “cellular component” into 16 subcategories and “molecular function” into 14 subcategories. As shown in Figure 4A, the largest subcategory found in the “biological process” group was “metabolic process” which comprised 32.1% of the unigenes in the subcategory. By applying COG platform, we obtained 18,394 sequences involved in COG classification, which were grouped into 25 categories (Figure 4B). Among the 25 COG categories, “general function prediction only” was the most populated group (17.72%) followed by “carbohydrate transport and metabolism” (8.27%) and “amino acid transport and metabolism” (7.59%). 

Furthermore, the Kyoto Encyclopedia of Genes and Genomes (KEGG) database was used to identify the biological pathways in *A. flavus*. A total of 12,232 annotated unigenes were grouped to 108 KEGG pathways (Table S1). The pathways with the most representation among the unique sequences were involved in metabolic pathways (28.78%, 3520), biosynthesis of secondary metabolites (12.61%, 1543) and starch and sucrose metabolism (4.85%, 593). As expected, most unigenes belong to metabolic pathways because of their involvement in the maintenance of basic biological processes of *A. flavus*. The *A. flavus* genome sequence contains remarkable enzymatic genes associated with secondary metabolite synthesis [2,30], which intimates it has the capacity to express more unigenes for biosynthesis of secondary metabolites under specific conditions. It has been documented previously that *A. flavus* produces numerous hydrolyses [31], including α-amylase precursor, α-amylase A precursor, α-L-arabinofuranosidase precursor, β-galactosidase, catalase (A and B), glutaminase A and α-mannosidase, which are believed to be important for fungal utilization of starch-rich. These results are in full agreement with the KEGG annotations of the unigenes.

### 3.5. Identification and Analysis of DEGs 

**Figure 5:**
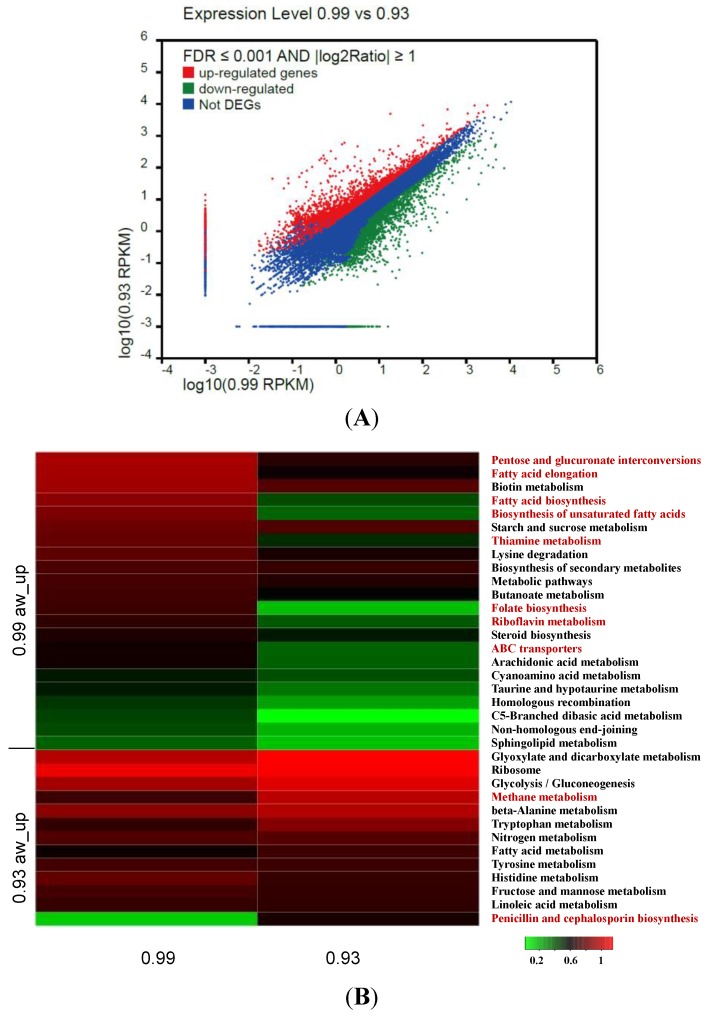

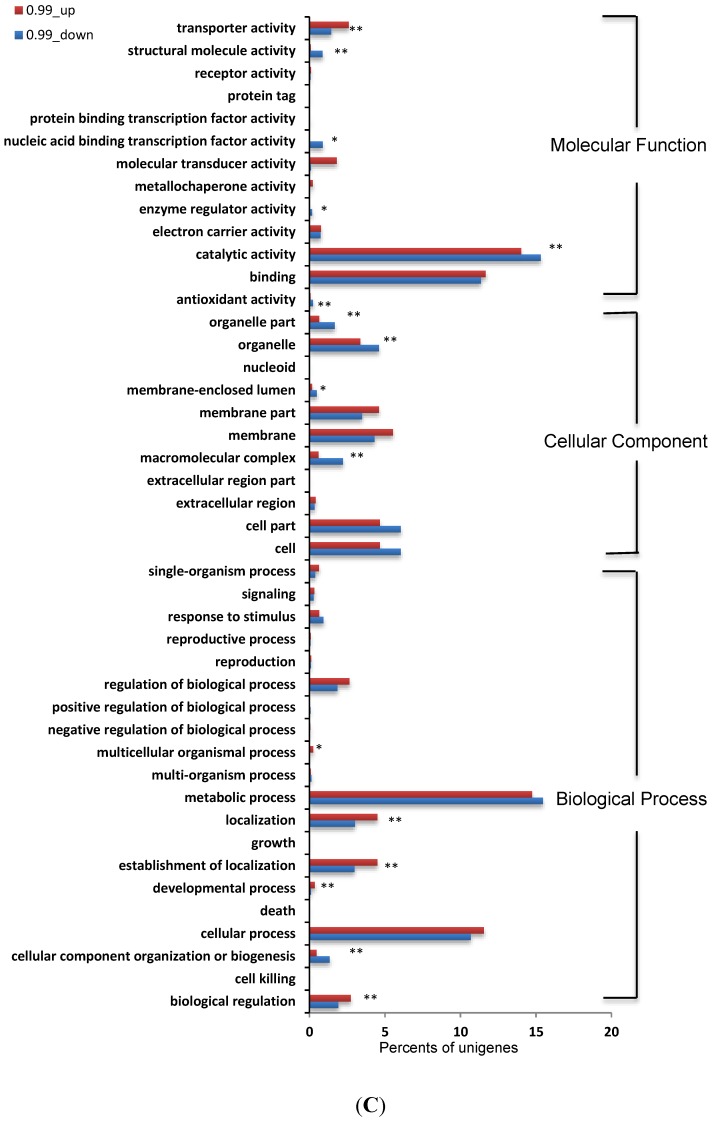
The different expression level of unigenes under different treatments. (**A**) Scatter plot of total unigenes from the *A. flavus* transcriptome; (**B**) KEGG annotation of DEGs. The heatmap shows 35 of 100 annotated pathways of DEGs between 0.99 a_w__up and 0.93 a_w__up. Among the 35 pathways, 19 pathways were up-regulated in 0.99 a_w_ treatment, and the rest of the pathways showed up-regulated expression in 0.93 a_w_ treatment. Different colors represent different expression level of a particular metabolic pathway during the two treatments. Green color represents down-regulated expression and red color represents up-regulated expression. Each row represents a differentially expressed metabolic pathway. The data used to construct this heatmap was based on the log10 value of the RPKM values of all unigenes relating to a particular metabolic pathway in 0.99 a_w_ or 0.93 a_w_ treatment. The top ten hits were shown with red words; (**C**) The gene ontology annotation of DEGs. Asterisks indicate a significant overrepresentation of functional categories compared to the functional categories of 3010 present genes (* *p* < 0.05; ** *p* < 0.01).

To identify the differences of molecular response between 0.99 a_w_ and 0.93 a_w_ treatments, gene expression levels were calculated using the RPKM method [22]. Based on RPKM values, out of 23,320 unigenes, 5362 differentially expressed unigenes (with *p* < 0.05, FDR ≤ 0.001, |log_2_Ratio| ≥ 1) were identified (Figure 5A). Among them, 3156 and 2206 genes displayed up-regulation under 0.99 a_w_ and 0.93 a_w_ treatments, respectively. All of the differentially expressed sequences were subjected to GO analysis, and the number of unigenes with GO annotations in 0.99 a_w_ DEGs (1714) was more than that of 0.93 a_w_ (1296). As shown in Figure 5C, the GO terms “transporter activity”, “localization”, “establishment of localization”, and “biological regulation” were significantly over-represented at the transcriptional level at 0.99 a_w_ compared with the 0.93 a_w_. In contrast, the GO categories “structural molecule activity”, “catalytic activity”, “nucleic acid binding transcription factor activity”, “organelle part”, “organelle”, “membrane-enclosed lumen”, “macromolecuar complex”, and “cellular component organization or biogenesis” were expressed at high levels under 0.93 a_w_ conditions, demonstrating that these factors play a pivotal role in adapting to water stress which is agreement with the results obtained by Abdel-Hadi* et al.* (2012) [16]. 

To study the function of DEGs, KEGG metabolic pathways analysis was performed by initially aligning unigenes with sequences from GenBank. Among 2721 DEGs, 1516 annotated unigenes up-expressed in 0.99 a_w_ conditions, and 1205 genes up-expressed in 0.93 a_w_ conditions were grouped into 108 known metabolic or signaling pathway classes (Table S2). Although the pathway distributions of these up-expressed genes in both 0.99 a_w_ and 0.99 a_w_ were almost in accordance with each other, more genes displayed at least two-fold up-regulation in 0.99 a_w_ conditions (Figure 5B). For example, more genes related to fatty acid metabolism such as “fatty acid biosynthesis”, “biosynthesis of unsaturated fatty acids” and “fatty acid elongation” displayed high transcriptional activity at 0.99 a_w_. Aflatoxins are known to begin with norsolorinic acid, which is synthesized* in vivo* by a specialized pair of fatty acid synthases (FAS-1 and FAS-2) and a separately transcribed polyketide synthase (PKS-A) [32]. 

### 3.6. Analysis of DEGs Involved in Aflatoxin Biosynthesis

To evaluate the effect of water activity on the regulation of aflatoxin biosynthesis, we used the sequence information of 33 candidate genes provided by NCBI to identify the putative aflatoxin biosynthesis genes in the *A. flavus* transcriptomes [15]. As shown in Table 2, a number of different expression genes related to aflatoxin biosynthesis in response to water stress were identified. Among the 33 candidate genes identified in the transcriptome of *A. flavus*, 16 genes were up-regulated more than twofold in 0.99 a_w_ conditions compared with 0.93 a_w_ conditions. Several genes coding for aflatoxin biosynthesis were significant differences between the two regimes. For example, *aflF*, *aflU* and *aflG* all have more than 10-fold transcriptional changes in 0.99 a_w_ relative to 0.93 a_w_ conditions. The gene *aflF* also named *norB*, shares 68% amino acid similarity to an aryl alcohol dehydrogenase encoded by an *aflE* (*norA*) gene, which is putatively involved in the conversion of NOR to AVN [33]. An additional gene, *aflD* (*nor-1*), was identified in the aflatoxin gene cluster encoding a ketoreductase that is capable of converting NOR to AVN [34]. Therefore, the presence of one of them was enough to catalyze NOR to AVN [35], which may help explain the phenomenon that only *aflF* up-regulated more than 2-folds. The gene *aflU* encodes a polypeptide of 498 amino acids, which has a typical heme-binding motif of cytochrome P450 monooxygenase [35]. Based on sequence analysis and the enzymatic requirement for G-group toxin biosynthesis, this gene is most likely involved in G-group toxin formation in aflatoxin biosynthesis [33]. A previous expression study revealed that its transcript was detected only under aflatoxin-conducive conditions and not on non-conducive conditions [35], which is in a good agreement with our findings. The gene *aflG* encodes a cytochrome P450 monooxygenase that converts AVN to HAVN [36]. A striking finding that *aflG*/*aflL* is contiguous only in the cluster of section *Flavi* species suggested *aflG*/*aflL* either was recruited from other genomic locations or reorganization of cluster genes from a sterigmatocystin ancestor [37]. The gene *aflNa* (*hypD*), first reported by expressed sequence tag data, has been predicted to encode a small integral membrane protein and suggested to affect both development and secondary metabolism of *Aspergillus* [38]. Interestingly, among the five most highly up-regulated genes, *aflF*, *aflU* and *aflT* genes are adjacent and located on the very end of the gene cluster, whereas *aflG* and *aflNa* are located next to each other in the middle of the gene cluster. Therefore, the gene* aflF* could be related to turning on/off aflatoxin pathway gene expression, and on chromosomal location these gene may be responsive to the environmental queue of water activity. The gene *aflR* is a Zn_2_Cys_6_-type transcription factor that is believed to be necessary for regulating most of the genes in the aflatoxin gene cluster in *A. flavus* [38], and they demonstrated that water activity had a significant effect on *aflR* transcription at lower a_w_ (0.90) compared with higher a_w_ (0.99) [39]. Curiously, the expression of this gene did not display somewhat difference even though strains were removed to a favor aflatoxin-producing regimes.

### 3.7. Analysis of DEGs Involved in Development

The control of secondary metabolism in fungi is often coordinated to fungal growth and development [40]. To further explore potential DEGs involved in aflatoxin biosynthesis in *A. flavus*, we analyzed 69 annotated sequences for the genes involved in development [41]. We found that the transcriptional patterns of most genes involved in development were down-regulated when *A. flavus* was treated with a lower water activity. For instance, *flbC* encoding C_2_H_2_ transcription factor, which is involved in asexual development, sexual development and germination, decreased its RPKM value from 135.08 to 5.67 (Table 3). In wild-type colonies, FlbC localizes in the nuclei of vegetative hyphae and in conidiophores, activates *brlA*, *abaA*, and *vosA* but not *wetA* [42]. Apart from *flbC*, four *flb* genes, *flbA*, *flbB*,* flbD* and *flbE* were taken into account in present study. *FlbA* encodes an RGS domain protein, which negatively regulates vegetative growth signaling [43]. *FlbB* encodes a fungal specific bZIP-type transcription factor, which is located within the cytoplasm at the hyphal apex during early vegetative growth and involved in asexual development [44]. FlbD, a c-Myb transcription factor, is uniquely involved in both asexual and sexual differentiation in *A. nidulans* [45]. FlbE localized at hyphal tips, which may protect FlbB from proteolytic degradation [46]. Although the *flb* genes are conserved in *A. fumigatus*, *A. oryzae* and *A. nidulans* [41], only *flbC* was un-regulated in the current study. This inconsistency may be explained by FlbC acting in a pathway parallel to that of other *flb* genes. Alternatively, the promoter-binding regions of FlbC and FlbB/FlbD may overlap [47].

**Table 2:** Expression profiling of *A. flavus* genes involved in aflatoxin biosynthesis.

Gene	ref_ID	Function	0.99_RPKM	0.93_RPKM	Log_2_ (0.99_RPKM/0.93_RPKM)	Changes (*)
*aflF*	XP_002379954	Dehydrogenase	5.2	0.1	6.3	Up
*aflU*	XP_002379953	P450 monooxygenase	16.3	1.1	3.9	Up
*aflG*	XP_002379937	Cytochrome P450 monooxygenase	50.6	4.2	3.6	Up
*aflNa*	XP_002379938	Hypothetical protein	121.4	17.5	2.8	Up
*aflT*	XP_002379952	Transmembrane protein	10.4	1.7	2.6	Up
*aflQ*	XP_002379931	Cytochrome P450 monooxigenase	163.3	28.3	2.5	Up
*aflJ*	XM_002379902	Esterase	6.1	1.3	2.2	Up
*aflI*	XP_002379934	Cytochrome P450 monooxygenase	1.1	0.3	1.7	Up
*aflMa*	XP_002379940	Hypothetical protein	11.9	4.1	1.5	Up
*aflYb*	XP_002379924	Putative hexose transporter	5.9	2.2	1.4	Up
*aflYd*	XP_002379922	Sugar regulator	2.8	1.1	1.4	Up
*aflX*	XP_002379927	Monooxygenase oxidase	6.4	2.4	1.4	Up
*aflB*	XP_002379947	Fatty acid synthase beta subunit	149.9	72.2	1.1	Up
*aflW*	XP_002379928	Monooxygenase	3.2	1.6	1.0	Up
*aflY*	XP_002379926	Hypothetical protein	4.4	2.2	1.0	Up
*aflL*	XP_002379936	P450 monooxygenase	4.6	2.4	0.9	
*aflN*	XP_002379939	Monooxygenase	3.1	1.7	0.9	
*aflV*	XP_002379929	Cytochrome P450 monooxygenase	2.1	1.2	0.8	
*aflE*	XP_002379942	NOR reductase dehydrogenase	6.5	4.1	0.7	
*aflM*	XP_002379941	Ketoreductase	14.0	10.8	0.4	
*aflYa*	XP_002379925	NADH oxidase	2.1	1.6	0.4	
*aflS*	XP_002379945	Pathway regulator	0.6	0.5	0.3	
*aflP*	XP_002379932	*O*-methyltransferase A	4.7	4.2	0.2	
*aflK*	XP_002379930	VERB synthase	11.5	11.2	0.0	
*aflR*	XM_002379905	Transcription activator	0.1	0.1	0.0	
*aflLa*	XP_002379935	Hypothetical protein	1.4	1.6	−0.1	
*aflO*	XP_002379933	*O*-methyltransferase B	6.7	7.7	−0.2	
*aflH*	XP_002379944	Short chain alcohol dehydrogenase	4.4	5.5	−0.3	
*aflA*	XP_002379948	Fatty acid synthase alpha subunit	4.7	6.0	−0.3	
*aflCa*	XP_002379950	Hypothetical protein	0.4	0.6	−0.4	
*aflD*	XP_002379949	Reductase	0.4	0.6	−0.4	
*aflYc*	XP_002379923	Glucosidase	19.9	26.2	−0.4	
*aflC*	XP_002379951	Polyketide synthase	4.8	7.7	−0.7	

^(^*^)^ Log_2_ (0.99_RPKM/0.93_RPKM) ≥1 indicate up-regulated expression while Log_2_ (0.99_RPKM/0.93_RPKM) ≤−1 indicate down-regulated expression.

**Table 3:** Expression profiling of *A. flavus* genes involved in development.

Gene	ref_ID	Function	0.99_RPKM	0.93_RPKM	Log_2_ (0.99_RPKM/0.93_RPKM)	Changes ^(^*^)^
*rgsA*	gi|259484767|tpe|CBF81270.1|	G protein regulator	ND	ND		
*nsdD*	gi|259485893|tpe|CBF83303.1|	DNA binding protein	ND	ND		
*flbC*	gi|259487830|tpe|CBF86815.1|	Putative zinc finger protein	135.1	5.7	4.6	Up
*cryA*	gi|40747330|gb|EAA66486.1|	Hypothetical protein	0.2	0	3.2	Up
*MAT1-1*	gi|259486330|tpe|CBF84081.1|	Mating type alpha box protein	1.1	0.1	3.2	Up
*gprB*	gi|34482020|tpg|DAA01795.1|	Pheromone receptor	9.9	1.7	2.5	Up
*abr1*	gi|6090821|gb|AAF03353.1|	Brown 1	2.8	0.7	1.9	Up
*brnA*	gi|134081843|emb|CAK42098.1|	Unnamed protein product	2.8	0.7	1.9	Up
*MAT1-2*	gi|259482427|tpe|CBF76901.1|	Mating type HMG-box protein	8.6	2.5	1.8	Up
*brlA*	gi|259488735|tpe|CBF88417.1|	Regulatory protein	7.7	2.3	1.8	Up
*lreA*	gi|259485576|tpe|CBF82714.1|	GATA-factor	6.6	2.9	1.2	Up
*stuA*	gi|259480005|tpe|CBF70741.1|	Cell pattern formation-associated protein	3.8	1.8	1.1	Up
*medA*	gi|259479562|tpe|CBF69898.1|	Medusa	6.1	3	1.1	Up
*tpsB*	gi|1488038|gb|AAB05869.1|	Trehalose-6-phosphate synthase	91.5	47.4	0.9	
*tpsA*	gi|3170246|gb|AAC18060.1|	Trehalose-6-phosphate synthase subunit 1	91.5	47.4	0.9	
*steA*	gi|259487683|tpe|CBF86542.1|	Transcription factor	25	15	0.8	
*gprA*	gi|34482022|tpg|DAA01796.1|	Pheromone receptor	4.4	2.8	0.7	
*gprD*	gi|259485627|tpe|CBF82810.1|	Integral membrane protein	11.3	7	0.7	
*lreB*	gi|259481867|tpe|CBF75789.1|	GATA-factor	76.3	51.6	0.6	
*rosA*	gi|259484624|tpe|CBF81007.1|	Repressor of sexual development	*47.1*	30.7	0.6	
*nosA*	gi|259487198|tpe|CBF85681.1|	NosA protein	47.1	30.7	0.6	
*wetA*	gi|259487296|tpe|CBF85858.1|	Regulatory protein	5.3	3.6	0.6	
*phnA*	gi|259489726|tpe|CBF90234.1|	Phosducin-like protein	33.1	21.7	0.6	
*arp2*	gi|6090729|gb|AAF03314.1|	Tetrahydroxynaphthalene reductase	14	10.8	0.4	
*kapA*	gi|259487521|tpe|CBF86262.1|	Karyopherin alpha	281.4	221.4	0.4	
*arp1*	gi|2555060|gb|AAC49843.1|	Scytalone dehydratase	63	52.6	0.3	
*nsdC*	gi|259481122|tpe|CBF74364.1|	NSDC	8	6.5	0.3	
*schA*	gi|259481151|tpe|CBF74417.1|	CAMP-dependent protein kinase-like	2.9	2.4	0.3	
*abaA*	gi|167998|gb|AAA33286.1|	AbaA protein	6	5.7	0.1	
*pkaA*	gi|259479481|tpe|CBF69742.1|	CAMP-dependent protein kinase	23.3	21.1	0.1	
*mpkB*	gi|259481736|tpe|CBF75537.1|	Mitogen-activated protein kinase	26.4	25.2	0.1	
*flbB*	gi|259483861|tpe|CBF79600.1|	bZIP-type transcription factor	48	43.4	0.1	
*fphA*	gi|259486541|tpe|CBF84471.1|	Phytochrome	10.1	9	0.1	
*steC*	gi|259487662|tpe|CBF86503.1|	MAPKK kinase	17.3	16.3	0.1	
*veA*	gi|259488644|tpe|CBF88249.1|	Mutant VeA1 protein	32.2	30.7	0.1	
*laeA*	gi|259488911|tpe|CBF88745.1|	Methyltransferase	19.3	17.5	0.1	
*flbE*	gi|259489004|tpe|CBF88918.1|	Putative uncharacterized protein	54.4	51.4	0.1	
*flbA*	gi|259479939|tpe|CBF70620.1|	Developmental regulator	18.4	18.4	0.0	
*gpgA*	gi|259486344|tpe|CBF84107.1|	G protein gamma subunit	248.6	247	0.0	
*sfaD*	gi|259489728|tpe|CBF90238.1|	G-protein beta subunit	78	77.2	0.0	
*abr2*	gi|6090815|gb|AAF03349.1|	Brown 2	6.8	7.5	−0.2	
*gpaA*	gi|27524346|emb|CAC81704.1|	GMP binding protein alpha subunit	43.8	47.3	−0.2	
*gpaB*	gi|27524350|emb|CAC81805.1|	GMP binding protein alpha subunit	1.6	1.9	−0.2	
*pkaB*	gi|67537094|ref|XP_662321.1|	Hypothetical protein	0.6	0.7	−0.2	
*gaoC*	gi|83773752|dbj|BAE63877.1|	Unnamed protein product	43.8	47.3	−0.2	
*yA*	gi|259480215|tpe|CBF71142.1|	Laccase-1 Precursor	6.8	7.5	−0.2	
*ganB*	gi|259488687|tpe|CBF88328.1|	G protein alpha subunit	1.6	1.9	−0.2	
*fadA*	gi|259489081|tpe|CBF89057.1|	GMP binding protein subunit alpha	43.8	47.3	−0.2	
*velB*	gi|259489398|tpe|CBF89638.1|	VelB	15.1	16.6	−0.2	
*flbD*	gi|259489501|tpe|CBF89824.1|	Putative uncharacterized protein	9.9	11.4	−0.2	
*alb1*	gi|3136092|gb|AAC39471.1|	Polyketide synthase	13.9	17.4	−0.3	
*chsC*	gi|4519181|dbj|BAA75501.1|	Chitin synthase	8.8	11	−0.3	
*fwnA*	gi|134078436|emb|CAL00851.1|	Unnamed protein product	13.9	17.4	−0.3	
*sfgA*	gi|259480894|tpe|CBF73944.1|	SfgA	0.8	0.9	−0.3	
*treB*	gi|2827392|gb|AAB99831.1|	Neutral trehalase	43.2	62.1	−0.5	
*pptA*	gi|134080185|emb|CAK46165.1|	Unnamed protein product	38.3	54.6	−0.5	
*cyaA*	gi|259481514|tpe|CBF75105.1|	Adenylate cyclase	1.6	2.4	−0.5	
*vosA*	gi|259487318|tpe|CBF85898.1|	VosA	4.3	6.2	−0.5	
*ppoB*	gi|259479464|tpe|CBF69709.1|	Fatty acid oxygenase	15.4	27.7	−0.7	
*ppoC*	gi|259482096|tpe|CBF76249.1|	Fatty acid oxygenase	15.4	27.7	−0.7	
*ppoA*	gi|259487326|tpe|CBF85912.1|	Fatty acid oxygenase	15.4	27.7	−0.7	
*rasA*	gi|259489610|tpe|CBF90024.1|	Ras-like protein	1.1	1.8	−0.7	
*chsA*	gi|465390|dbj|BAA04806.1|	Chitin synthase	8.7	19.6	−1.3	Down
*fluG*	gi|259482332|tpe|CBF76713.1|	FluG	11.8	34.5	−1.7	Down
*chiB*	gi|259485098|tpe|CBF81882.1|	Class V chitinase	0.1	0.1	−1.7	Down
*rolA*	gi|28875529|dbj|BAC65230.1|	Hydrophobin putative	1.5	6.9	−2.3	Down
*rodB*	gi|70996676|ref|XP_753093.1|	Conidial hydrophobin	1.5	6.9	−2.3	Down
*rodA*	gi|259482991|tpe|CBF77990.1|	Rodlet protein	1.5	6.9	−2.3	Down
*ganA*	gi|259485962|tpe|CBF83426.1|	G protein alpha subunit homolog	ND	ND		

^(^*^)^ Log_2_ (0.99_RPKM /0.93_RPKM) ≥1 indicate up-regulated expression while Log_2_ (0.99_RPKM/0.93_RPKM) ≤−1 indicate down-regulated expression. ND means no detection.

## 4. Conclusions

*Aspergillus flavus* is an imperfect filamentous fungal pathogen causing diseases of many agricultural crops, such as maize, cotton, and peanuts, as well as tree nuts [48]. In the current work, a transcriptome database of *A. flavus* was constructed. From the two different treatments (0.99 a_w_ and 0.93 a_w_), we identified differentially expressed genes by transcriptome analysis and found that numerous metabolic pathways related to biosynthesis were significantly over-expressed when treated with 0.99 a_w_, especially in the biosynthesis of aflatoxin in *A. flavus*. During treatment with 0.99 a_w_, unigenes involved in development, such as *flbC*, were significantly up-regulated. The relationship between the aflatoxin biosynthesis pathway and development of *A. flavus* is complex and further analytical work is required. Moisture is an important regime factor for fungi growth and mycotoxin production, but little transcription level information is available at present; therefore, our transcriptome provides a resource for further studies examining water activity, and fungi growth and aflatoxin production. Collectively, this study opens the way to future studies analyzing the effect of water activity on other fungi physiology.

## Supplementary Materials

Supplementary materials can be accessed at: http://www.mdpi.com/2072-6651/6/11/3187/s1.

## Supplementary Files

Supplementary File 1
