# Supplementary material for: RNA-Seq-Based Transcriptome Analysis of Aflatoxigenic Aspergillus flavus in Response to Water Activity
Source: Toxins (Basel). 2014 Nov 21;6(11):3187–207. doi: 10.3390/toxins6113187 (PMC4247253; doi:10.3390/toxins6113187)
Supplement: Supplementary File 1 [file toxins-06-03187-s001.pdf]

Supplementary Information

Table S1. Annotation of all-unigenes for the *A. flavus* transcriptome by KEGG classification.

	**Pathway**	**All genes with pathway annotation (12232)**	**Pathway ID**
1	Metabolic pathways	3520 (28.78%)	ko01100
2	Biosynthesis of secondary metabolites	1543 (12.61%)	ko01110
3	Starch and sucrose metabolism	593 (4.85%)	ko00500
4	RNA transport	477 (3.9%)	ko03013
5	Amino sugar and nucleotide sugar metabolism	432 (3.53%)	ko00520
6	Purine metabolism	410 (3.35%)	ko00230
7	MAPK signaling pathway—yeast	407 (3.33%)	ko04011
8	Cell cycle—yeast	350 (2.86%)	ko04111
9	Protein processing in endoplasmic reticulum	313 (2.56%)	ko04141
10	Meiosis—yeast	299 (2.44%)	ko04113
11	Glycerophospholipid metabolism	284 (2.32%)	ko00564
12	RNA degradation	280 (2.29%)	ko03018
13	Ribosome	279 (2.28%)	ko03010
14	Pyrimidine metabolism	274 (2.24%)	ko00240
15	Endocytosis	269 (2.2%)	ko04144
16	Spliceosome	267 (2.18%)	ko03040
17	Lysine degradation	240 (1.96%)	ko00310
18	Peroxisome	239 (1.95%)	ko04146
19	Butanoate metabolism	219 (1.79%)	ko00650
20	Ribosome biogenesis in eukaryotes	212 (1.73%)	ko03008
21	Tyrosine metabolism	208 (1.7%)	ko00350
22	Fructose and mannose metabolism	207 (1.69%)	ko00051
23	Tryptophan metabolism	198 (1.62%)	ko00380
24	mRNA surveillance pathway	197 (1.61%)	ko03015
25	Glycine, serine and threonine metabolism	188 (1.54%)	ko00260
26	Steroid biosynthesis	168 (1.37%)	ko00100
27	Arginine and proline metabolism	166 (1.36%)	ko00330
28	Cyanoamino acid metabolism	164 (1.34%)	ko00460
29	Fatty acid metabolism	156 (1.28%)	ko00071
30	Glutathione metabolism	152 (1.24%)	ko00480
31	Galactose metabolism	149 (1.22%)	ko00052
32	Glycolysis / Gluconeogenesis	142 (1.16%)	ko00010
33	Ubiquitin mediated proteolysis	141 (1.15%)	ko04120
34	Phenylalanine metabolism	139 (1.14%)	ko00360
35	ABC transporters	138 (1.13%)	ko02010
36	Linoleic acid metabolism	138 (1.13%)	ko00591
37	Oxidative phosphorylation	136 (1.11%)	ko00190
38	Histidine metabolism	133 (1.09%)	ko00340
39	Nitrogen metabolism	124 (1.01%)	ko00910
40	Glycerolipid metabolism	119 (0.97%)	ko00561
41	RNA polymerase	117 (0.96%)	ko03020
42	Pyruvate metabolism	114 (0.93%)	ko00620

Table S1. *Cont.*

	Pathway	All genes with pathway annotation (12232)	Pathway ID
43	Homologous recombination	113 (0.92%)	ko03440
44	Fatty acid biosynthesis	107 (0.87%)	ko00061
45	Cysteine and methionine metabolism	107 (0.87%)	ko00270
46	Pentose and glucuronateinterconversions	106 (0.87%)	ko00040
47	Nucleotide excision repair	104 (0.85%)	ko03420
48	Methane metabolism	103 (0.84%)	ko00680
49	Non-homologous end-joining	103 (0.84%)	ko03450
50	Phagosome	103 (0.84%)	ko04145
51	Alanine, aspartate and glutamate metabolism	101 (0.83%)	ko00250
52	Aminoacyl-tRNA biosynthesis	100 (0.82%)	ko00970
53	DNA replication	97 (0.79%)	ko03030
54	Propanoate metabolism	97 (0.79%)	ko00640
55	Valine, leucine and isoleucine degradation	93 (0.76%)	ko00280
56	Basal transcription factors	91 (0.74%)	ko03022
57	N-Glycan biosynthesis	90 (0.74%)	ko00510
58	Inositol phosphate metabolism	85 (0.69%)	ko00562
59	Pantothenate and CoA biosynthesis	80 (0.65%)	ko00770
60	Biosynthesis of unsaturated fatty acids	77 (0.63%)	ko01040
61	Citrate cycle (TCA cycle)	77 (0.63%)	ko00020
62	Taurine and hypotaurine metabolism	76 (0.62%)	ko00430
63	Glycosylphosphatidylinositol(GPI)-anchor biosynthesis	74 (0.6%)	ko00563
64	Glyoxylate and dicarboxylate metabolism	74 (0.6%)	ko00630
65	Arachidonic acid metabolism	73 (0.6%)	ko00590
66	Phenylalanine, tyrosine and tryptophan biosynthesis	71 (0.58%)	ko00400
67	Base excision repair	70 (0.57%)	ko03410
68	Various types of N-glycan biosynthesis	68 (0.56%)	ko00513
69	Ether lipid metabolism	68 (0.56%)	ko00565
70	Sphingolipid metabolism	64 (0.52%)	ko00600
71	beta-Alanine metabolism	64 (0.52%)	ko00410
72	Valine, leucine and isoleucine biosynthesis	62 (0.51%)	ko00290
73	Regulation of autophagy	62 (0.51%)	ko04140
74	Ubiquinone and other terpenoid-quinone biosynthesis	61 (0.5%)	ko00130
75	Pentose phosphate pathway	59 (0.48%)	ko00030
76	Mismatch repair	58 (0.47%)	ko03430
77	Riboflavin metabolism	58 (0.47%)	ko00740
78	Porphyrin and chlorophyll metabolism	57 (0.47%)	ko00860
79	Lysine biosynthesis	57 (0.47%)	ko00300
80	Nicotinate and nicotinamide metabolism	57 (0.47%)	ko00760
81	Proteasome	57 (0.47%)	ko03050
82	Phosphatidylinositol signaling system	54 (0.44%)	ko04070
83	Folate biosynthesis	50 (0.41%)	ko00790

Table S1. *Cont.*

	Pathway	All genes with pathway annotation (12232)	Pathway ID
84	SNARE interactions in vesicular transport	50 (0.41%)	ko04130
85	Terpenoid backbone biosynthesis	45 (0.37%)	ko00900
86	One carbon pool by folate	42 (0.34%)	ko00670
87	Sulfur metabolism	42 (0.34%)	ko00920
88	Sulfur relay system	41 (0.34%)	ko04122
89	Penicillin and cephalosporin biosynthesis	33 (0.27%)	ko00311
90	Synthesis and degradation of ketone bodies	32 (0.26%)	ko00072
91	Fatty acid elongation	29 (0.24%)	ko00062
92	Selenocompound metabolism	26 (0.21%)	ko00450
93	Vitamin B6 metabolism	25 (0.2%)	ko00750
94	Protein export	24 (0.2%)	ko03060
95	Biotin metabolism	24 (0.2%)	ko00780
96	Other glycan degradation	23 (0.19%)	ko00511
97	alpha-Linolenic acid metabolism	21 (0.17%)	ko00592
98	Ascorbate and aldarate metabolism	21 (0.17%)	ko00053
99	Thiamine metabolism	21 (0.17%)	ko00730
100	Natural killer cell mediated cytotoxicity	16 (0.13%)	ko04650
101	Circadian rhythm—mammal	13 (0.11%)	ko04710
102	Other types of O-glycan biosynthesis	12 (0.1%)	ko00514
103	Glycosaminoglycan degradation	11 (0.09%)	ko00531
104	Glycosphingolipid biosynthesis—globo series	11 (0.09%)	ko00603
105	C5-Branched dibasic acid metabolism	9 (0.07%)	ko00660
106	Lipoic acid metabolism	7 (0.06%)	ko00785
107	Caffeine metabolism	7 (0.06%)	ko00232
108	D-Arginine and D-ornithine metabolism	4 (0.03%)	ko00472

Table S2. Annotation of DEGs for the *A. flavus* transcriptome by KEGG classification.

Pathway	0.93_up [(a)]	0.99_up [(b)]	Fold
Endocytosis	3	33	11
Folate biosynthesis	2	14	7
Valine, leucine and isoleucine biosynthesis	2	12	6
C5-Branched dibasic acid metabolism	1	4	4
Fatty acid biosynthesis	6	22	3.6
Biosynthesis of unsaturated fatty acids	6	21	3.5
Aminoacyl-tRNA biosynthesis	4	14	3.5
Ether lipid metabolism	3	9	3
Pantothenate and CoA biosynthesis	5	14	2.8
Biotin metabolism	3	8	2.6
Fatty acid elongation	3	8	2.6
Riboflavin metabolism	6	15	2.5
Ubiquitin mediated proteolysis	4	9	2.2
Purine metabolism	28	61	2.1
Sphingolipid metabolism	6	13	2.1
ABC transporters	15	32	2.1

Table S2. *Cont.*

Pathway	0.93_up [(a)]	0.99_up [(b)]	Fold
Non-homologous end-joining	9	19	2.1
Homologous recombination	10	21	2.1
Arachidonic acid metabolism	8	15	1.8
Taurine and hypotaurine metabolism	8	15	1.8
Steroid biosynthesis	23	43	1.8
Inositol phosphate metabolism	6	11	1.8
Pentose and glucuronateinterconversions	11	20	1.8
Meiosis—yeast	25	45	1.8
Lysine degradation	26	45	1.7
Alanine, aspartate and glutamate metabolism	11	18	1.6
Phagosome	5	8	1.6
Phenylalanine, tyrosine and tryptophan biosynthesis	5	8	1.6
Mismatch repair	4	6	1.5
Thiamine metabolism	4	6	1.5
Butanoate metabolism	29	42	1.4
Cyanoamino acid metabolism	25	36	1.4
Glycine, serine and threonine metabolism	28	40	1.4
Pentose phosphate pathway	7	10	1.4
Valine, leucine and isoleucine degradation	7	10	1.4
Spliceosome	15	21	1.4
Glutathione metabolism	16	21	1.3
Starch and sucrose metabolism	68	88	1.2
Peroxisome	21	27	1.2
Pyrimidine metabolism	22	28	1.2
Biosynthesis of secondary metabolites	188	238	1.2
Arginine and proline metabolism	19	24	1.2
Glycerophospholipid metabolism	28	35	1.2
Metabolic pathways	414	515	1.2
Pyruvate metabolism	10	12	1.2
Sulfur relay system	5	6	1.2
MAPK signaling pathway—yeast	42	50	1.1
Lysine biosynthesis	6	7	1.1
Cell cycle—yeast	31	35	1.1
Citrate cycle (TCA cycle)	8	9	1.1
RNA degradation	27	30	1.1
Various types of N-glycan biosynthesis	9	10	1.1
RNA polymerase	10	11	1.1
Linoleic acid metabolism	23	24	1.0
Galactose metabolism	16	16	1
Basal transcription factors	7	7	1
DNA replication	6	6	1
Ubiquinone and other terpenoid-quinone biosynthesis	4	4	1

Table S2. *Cont.*

Pathway	0.93_up [(a)]	0.99_up [(b)]	Fold
Ascorbate and aldarate metabolism	2	2	1
Natural killer cell mediated cytotoxicity	2	2	1
Amino sugar and nucleotide sugar metabolism	51	49	0.96
Histidine metabolism	20	19	0.95
Phenylalanine metabolism	20	19	0.95
Protein processing in endoplasmic reticulum	20	19	0.95
Fructose and mannose metabolism	32	30	0.93
RNA transport	31	29	0.93
Tyrosine metabolism	30	28	0.93
Propanoate metabolism	15	14	0.93
Glycerolipid metabolism	13	12	0.92
Nitrogen metabolism	20	18	0.90
Base excision repair	6	5	0.83
Ribosome biogenesis in eukaryotes	23	19	0.82
Phosphatidylinositol signaling system	5	4	0.80
mRNA surveillance pathway	19	15	0.78
Porphyrin and chlorophyll metabolism	8	6	0.75
Fatty acid metabolism	24	17	0.70
Oxidative phosphorylation	17	12	0.70
Nicotinate and nicotinamide metabolism	3	2	0.66
Tryptophan metabolism	36	23	0.63
One carbon pool by folate	8	5	0.62
beta-Alanine metabolism	13	8	0.61
Nucleotide excision repair	10	6	0.60
Penicillin and cephalosporin biosynthesis	12	7	0.58
Selenocompound metabolism	7	4	0.57
Glycolysis/Gluconeogenesis	23	12	0.52
Other glycan degradation	2	1	0.50
Methane metabolism	23	11	0.47
Ribosome	48	22	0.45
Sulfur metabolism	5	2	0.40
Terpenoid backbone biosynthesis	5	2	0.40
Cysteine and methionine metabolism	13	5	0.38
alpha-Linolenic acid metabolism	4	1	0.25
N-Glycan biosynthesis	12	2	0.16
Glyoxylate and dicarboxylate metabolism	15	0	0
Glycosylphosphatidylinositol(GPI)-anchor biosynthesis	5	0	0
Protein export	5	0	0
Lipoic acid metabolism	1	0	0
Proteasome	1	0	0
Caffeine metabolism	0	5	∞
Circadian rhythm—mammal	0	2	∞
Glycosaminoglycan degradation	0	2	∞

Table S2. *Cont.*

Pathway	0.93_up [a]	0.99_up [b]	Fold
Glycosphingolipid biosynthesis—globo series	0	1	∞
Glycosylphosphatidylinositol(GPI)-anchor biosynthesis	0	2	∞
Glyoxylate and dicarboxylate metabolism	0	9	∞
Regulation of autophagy	0	1	∞
SNARE interactions in vesicular transport	0	4	∞
Synthesis and degradation of ketone bodies	0	5	∞
Vitamin B6 metabolism	0	7	∞

[a] The total numbers of genes up-regulated in 0.93 a_w compared to in 0.99 a_w; [b] The total numbers of genes up-regulated in 0.99 a_w compared to in 0.93 a_w.
